# Eco-biofabrication of silver nanoparticles from *Azadirachta indica, Gymnema sylvestre,* and *Moringa oleifera* for lung cancer treatment

**DOI:** 10.1186/s43046-024-00252-0

**Published:** 2025-01-06

**Authors:** Tanya Muthu, Ravi Adusumalli, Sathish Kumar Vemuri, M. Indira Devi, P. Pavan Kumar, Rajkiran Reddy Banala, A. V. Gurava Reddy

**Affiliations:** 1 SMART, Sunshine Hospitals, Secunderabad, Telangana India; 2 Department of Biosciences, University of Oslo, Blindern, Oslo 0316 Norway; 3 Mahatma Gandhi University, Nalgonda District, Telangana India; 4 Translational Research Center, Asian Healthcare Foundation, Hyderabad, India

**Keywords:** Biofabrication, Silver nanoparticles (AgNPs), *Gymnema sylvestre*, *Moringa oleifera*, *Azadirachta indica*, A549 cell line, Lung cancer

## Abstract

**Introduction:**

Silver nanoparticles (AgNPs) derived from natural sources have garnered significant attention due to their unique properties and eco-friendly production methods. With lung cancer remaining a major global health issue, there is a continuous need for novel and effective therapeutic approaches beyond conventional treatments such as chemotherapy, immunotherapy, and targeted therapies.

**Objective:**

This study aims to synthesize AgNPs using plant extracts from *Gymnema sylvestre*, *Moringa oleifera*, and *Azadirachta indica* and to evaluate their anticancer activity, particularly their effects on gene expression in A549 lung cancer cells.

**Methods:**

AgNPs were synthesized using green chemistry techniques and characterized by X-ray diffraction (XRD) and Fourier-transform infrared spectroscopy (FTIR). Gene expression studies were performed to assess the impact of AgNPs on cancer-related genes such as VEGF and CYCLIN-D1. Cytotoxicity assays were conducted on A549 cells to determine the anticancer potential of the synthesized AgNPs compared to plant extracts alone.

**Results:**

XRD confirmed the formation of crystalline AgNPs, while FTIR indicated the presence of bioactive compounds interacting with the nanoparticles. Gene expression analysis revealed significant downregulation of VEGF and CYCLIN-D1, suggesting inhibitory effects on angiogenesis and cell cycle progression. The synthesized AgNPs exhibited potent cytotoxic activity against A549 cells, with enhanced efficacy compared to the leaf extracts alone.

**Conclusion:**

The study highlights the potential of AgNPs synthesized from medicinal plant extracts as promising candidates for lung cancer therapy. Their environmentally sustainable production, combined with their ability to target key cancer pathways, positions them as innovative and affordable therapeutic agents in the field of nanomedicine.

## Introduction

The application of nanotechnology in cancer treatment has gained significant momentum, with silver nanoparticles (AgNPs) being at the forefront due to their unique physicochemical properties and potent biological activities. Among the various synthesis methods, green synthesis using medicinal plants such as *Azadirachta indica*, *Gymnema sylvestre*, and *Moringa oleifera* has emerged as a promising approach [1–3]. This method not only aligns with the principles of sustainability but also enhances the biocompatibility and therapeutic potential of the nanoparticles. The green synthesis process utilizes the bioactive compounds present in these plants to reduce silver ions into nanoparticles, which can then be used in various biomedical applications, including cancer therapy [1–6].

The potential of AgNPs in cancer treatment is largely attributed to their ability to induce cytotoxic effects in cancer cells through mechanisms such as the generation of reactive oxygen species (ROS), DNA damage, and the induction of apoptosis [4, 5]. Additionally, the incorporation of phytochemicals from medicinal plants during the synthesis process has been shown to further enhance these therapeutic effects while reducing toxicity to normal cells, thereby improving overall biocompatibility [7–13].

Lung cancer, which remains one of the leading causes of cancer-related mortality worldwide, particularly benefits from such targeted nanotherapeutic strategies [14–16]. AgNPs synthesized using plant extracts have demonstrated promising potential in targeting lung cancer cells, offering a multi-faceted approach that includes direct cytotoxic effects and modulation of the tumor microenvironment [17–20]. This introduction sets the stage for a comprehensive review of recent advances in the green synthesis of AgNPs using medicinal plants and their potential applications in lung cancer therapy.

Nanotechnology has revolutionized multiple scientific fields, including medicine, by offering immense possibilities for innovation and transformative applications [21–24]. Within the realm of nanomaterials, silver nanoparticles (AgNPs) have attracted significant interest due to their exceptional physicochemical properties and wide-ranging antimicrobial and therapeutic capabilities [25–28]. The pursuit of sustainable and environmentally friendly methods for synthesizing nanomaterials has led to a renewed focus on the green synthesis of AgNPs, which combines the principles of green chemistry with the abundant phytochemical resources present in medicinal plants [29–32].

*Azadirachta indica* (Neem/AI), *Gymnema sylvestre* (GS/Madhunasini), and *Moringa oleifera* (MO/Moringa), stand out as three exceptional medicinal plants celebrated for their diverse bioactive compounds and extensive historical medicinal uses [1–3]. The utilization of plant extracts in AgNP synthesis provides a dual advantage: it helps alleviate the environmental challenges associated with conventional chemical synthesis methods while also capitalizing on the therapeutic capabilities of these plants for biomedical applications [27–31].

Lung cancer, a major factor in the global burden of cancer-related fatalities, remains a substantial challenge within the oncology domain [13]. While conventional treatments exhibit certain levels of effectiveness, they often bring about significant side effects and restrictions. This emphasizes the requirement for innovative, targeted, and biocompatible strategies in the treatment of lung cancer [7]. The ongoing investigation focuses on the sustainable synthesis of AgNPs through the utilization of *Azadirachta indica*, *Gymnema sylvestre*, and *Moringa oleifera*, aiming to establish a novel therapeutic intervention for lung cancer.

The present paper conducts a comprehensive examination of the green synthesis methodologies utilized, thoroughly characterizes the physicochemical properties of the synthesized AgNPs, and elucidates their potential biological activities. Furthermore, it delves into in vitro and in vivo studies to evaluate the anti-cancer efficacy of these AgNPs against lung cancer [20, 32–39]. This research aims to explore the potential of using AgNPs derived from medicinal plants as a safe and effective method for treating lung cancer. The investigation will focus on parameters such as cytotoxicity, induction of apoptosis, and molecular mechanisms, which will provide valuable insights into the feasibility of this approach.

To ascertain the scientific significance and originality of this study, we provide an extensive analysis of the existing literature on the green synthesis of nanoparticles, the anti-cancer properties exhibited by silver nanoparticles, and the therapeutic attributes of *Azadirachta indica*, *Gymnema sylvestre*, and *Moringa oleifera* in the context of cancer treatment. By adopting a multidisciplinary approach that integrates principles from nanotechnology, plant biology, and oncology, this research offers valuable insights that hold promise for the advancement of cancer therapeutics. The quest for eco-friendly and biocompatible nanoparticles synthesized from medicinal plants is an exciting and rapidly evolving frontier in the field of nanomedicine. This study contributes to this dynamic landscape by offering potential solutions for the significant health challenge that lung cancer presents globally.

## Materials and methods

### Leaf extract preparation

Leaves of *Azadirachta indica*, *Gymnema sylvestre*, and *Moringa oleifera* were sourced from local markets and air-dried at room temperature for 7 days. Once fully dried, the leaves were crushed into a fine powder. One gram of the powdered leaf material was then dissolved in 10 mL of distilled water, and the solution was left to sit at room temperature for 24 h.

### Synthesis of silver nanoparticles from leaf extracts

Prepare a solution of 1 mM Silver Nitrate (32814-Silver Nitrate extrapure, 99.5%, SRL, CAS: 7761–88-8), then mix 0.5 ml of leaf extract with 4.5 ml of the prepared solution, thoroughly mixing for 2–3 min. Incubate the mixture in a water bath for 30–45 min at 70–80 °C. The color change to reddish-brown indicates nanoparticle formation; store the mixture at 4–5 °C for future use [11].

### Purification of silver nanoparticles

Spin the solution at 6000 rpm for 60 min at 4 °C, discard the supernatant, and rinse the pellet twice with distilled water. Centrifuge again at 6000 rpm for 30 min, discard the supernatant, and air dry the pellet for 10 min. Reconstitute the pellet with 1 ml of double distilled water and store it at 4 °C for further use [11].

### Confirmation of silver nanoparticles by spectral analysis

Take 200 µL of reconstituted pellet mixture in a sterile 1.5 ml tube and add 800 µL of double distilled water. Measure the percentage of transmittance at 400–450 nm using double distilled water as a blank in the UV–visible spectrophotometer [11].

### Cell culture

A549 and CAL27 cells were cultured in Complete DMEM (Dulbecco’s Modified Eagle Medium, Gibco, Cat #0.11965118) media with 10% FBS (Fetal bovine serum, Gibco, Cat #. 10,091,155) and 1X Pen-Strep antibiotic (Gibxo, Cat 15,140,122) until reaching 80% confluency at 37 °C in a CO2 incubator with 5% humidity [12, 13].

### MTT assay

Seed 100 µL of 10^5^ cells/ml in 96-well plates. Incubate the plates in a 37 °C incubator with 5% CO_2_ and humidity. Treat the cells with 5 µg/ml, 10 µg/ml, and 20 µg/ml of leaf extracts and AGNPs of Moringa, Madhunasini, and Neem in triplicates. Untreated cells serve as control. Incubate the plates in a 37 °C incubator with 5% CO_2_ and humidity for 36 h. After incubation, wash the cells with 1X PBS, add 10 µL of MTT reagent (CyQUANT™ MTT Cell Viability Assay, Invitrogen™, Cat #. V13154) in the dark and incubate the cells at 37 °C for 2 h. Add 100 µL of DMSO (28580-Dimethylsulphoxide ACS, 99.9%, SRL, CAS: 67–68-5) to the wells. Read the plate at 570–650 nm. Calculate the percentage of cell viability using the formula: % cell viability = O.D of treated cells/O.D of untreated cells × 100 [12, 13].

### Cell death and apoptosis assay

To assess cell death and apoptosis, we used the Annexin V-FITC/PI Apoptosis Detection Kit (Invitrogen, Cat. # V13242). Cells were harvested from culture using trypsin–EDTA (Gibco, Cat. # 15400054) and washed with phosphate-buffered saline (PBS). The cells were then resuspended in a culture medium, counted, and adjusted to a concentration of 1 × 10^6^ cells/mL. The cell suspension was centrifuged at 300 × g for 5 min, and the supernatant was discarded. The cell pellet was resuspended in 100 μL of binding buffer, followed by the addition of 5 μL of Annexin V-FITC and 5 μL of propidium iodide (PI). After gentle mixing, the cells were incubated in the dark at room temperature for 15 min. Subsequently, 400 μL of binding buffer was added, and the samples were immediately analyzed using a flow cytometer. The analysis distinguished live cells (Annexin V − /PI −), early apoptotic cells (Annexin V + /PI −), and late apoptotic or necrotic cells (Annexin V + /PI +) based on fluorescence intensity. This method provided a reliable quantification of cell death and apoptosis in the sample [12, 13].

### Expression studies

RNA Isolation: A549 cells were cultured in 12-well culture plates and treated for 36 h with concentrations of 5 µg/ml, 10 µg/ml, and 20 µg/ml of AgNPs of Moringa, Neem, and Madhunasini, along with untreated cells as controls. After incubation, the media is aspirated, and the cells are washed with 1X PBS and trypsinized. The cells are transferred to labeled 15 ml tubes with complete media and centrifuged at 7500 RPM for 5 min. RNA isolation is performed following the protocol of the RNAisoPlus (TAKARA kit, Cat. # 9108/9109). The RNA pellet is dissolved in 50 µL of nuclease-free water and stored at − 20 °C [13].

### cDNA synthesis from RNA

Verso cDNA Kit (Thermo Scientific, Cat. # AB1453A) reagents were used to generate full-length cDNA from RNA. The reaction was set up as per the Verso cDNA kit protocol in a thermocycler. The amplified cDNA was stored at − 20 °C until further use [13].

### DNA amplification from cDNA

The cDNA prepared is used to amplify specific DNA using the GoTaq Green Master Mix Protocol (GoTaq qPCR master mix Kit-PROMEGA), using primer sequences against GAPDH, VEGF, and CYCLIN-D1 [12, 13].

### Primer sequences

Glyceraldehyde 3-phosphate dehydrogenase Primer Sequence: 5′-GGTCTCCAGAACATCA TCCCTGCGGTGTCGCT G TTGAAGTCAGAGG-3′ Cyclin kinase inhibitor Primer Sequence: 5′-GCTTCCTGCAAGAGTC GAATATTGGCTTCTCA A GATACCTG-3′ Vascular endothelial growth factor Primer Sequence: 5′-GTACCTCCACCATGCC AAGT-3′. Five microliter of amplified DNA was loaded onto a 1% agarose gel, run, and documented using a Gel documentation system. All the primers were synthesized and procured from Euorfins, Bangalore, India [13].

## Results

### Synthesis of silver nanoparticles

The process begins with the preparation of leaf extracts obtained from Neem, Madhunashini, and Moringa plants. These extracts undergo a reaction with silver nitrate (AgNO3) by combining a solution of silver nitrate with 0.5 ml of each leaf extract and heating the mixture in a boiling water bath for 30 to 40 min [15]. During this reaction, the silver ions from AgNO3 interact with compounds present in the leaf extracts. As the reaction progresses, a color change from yellow to reddish-brown indicates the formation of silver nanoparticles. Once the reaction is complete, the solution is cooled to 4 °C and centrifuged at 6000 rpm for 1 h to separate the nanoparticles from the reaction mixture [15]. Pellets of silver nanoparticles are then formed in the Eppendorf tubes containing the leaf extract solutions after centrifugation. This method harnesses the reducing and stabilizing agents inherent in the leaf extracts to synthesize silver nanoparticles. The specific compounds responsible for the reduction and stabilization may vary depending on the plant species used. Silver nanoparticles produced through this green synthesis approach find diverse applications in fields such as medicine, catalysis, and electronics, owing to their unique properties and environmentally friendly synthesis process [21–26].

### Analysis of UV–visible spectroscopy

Pure Ag + ions were observed undergoing bio-reduction by periodically sampling 0.5 ml aliquots of the suspension and analyzing the resulting diluents using UV–Vis spectroscopy. The UV–Vis spectra of the generated silver nanoparticles were examined throughout the bio-reduction process at room temperature using a UV–visible spectrometer. The progression of the reaction between metal ions and leaf extracts was tracked by analyzing the UV–visible spectra of Ag nanoparticles in an aqueous solution [15, 29–35].

**Fig. 1:**
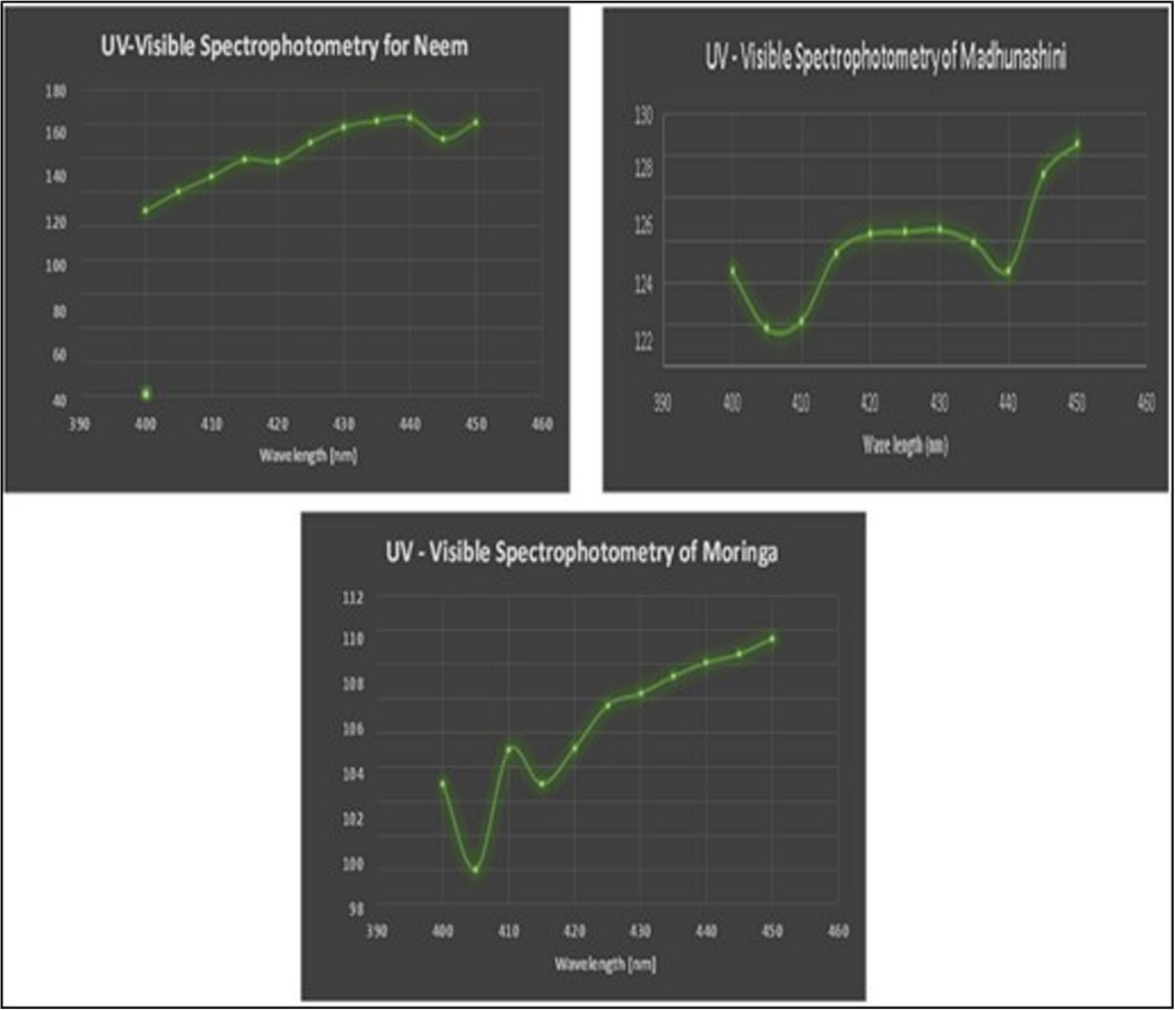
Graph UV–visible spectrophotometry of Neem, Madhunasini and Moringa

The above table and figure present the transmittance percentage and wavelength (nm), confirming the synthesis and presence of nanoparticles through the transmittance values of Neem, Madhunasini, and Moringa (Tables 1, 2, 3 and Fig. 1).

**Table 1:** Transmittance (%) studies of Neem, Madhunasini and Moringa AgNPs by UV—Visible Spectrophotometry

Wavelength (nm)	Transmittance (%)		
	***Azadirachta indica*/Neem**	***Gymnema sylvestre*/Madhunasini**	***Moringa oleifera*/Moringa**
400	109	122.5	101
405	120	119.8	96
410	129	120.2	103
415	138.9	123.4	101
420	138	124.3	103.1
425	149	124.4	105.6
430	158	124.5	106.3
435	161.9	123.9	107.3
440	163.7	122.6	108.1
445	151	127.1	108.6
450	161	128.6	109.5

**Table 2:** Depict the peaks and their respective functional groups

*Azadirachta indica* (Neem/AI)		*Gymnema sylvestre* (Madhunasini/GS)		*Moringa oleifera* (Moringa/MO)	
Frequencies	**Functional groups**	**Frequencies**	**Functional groups**	**Frequencies**	**Functional groups**
3743.83	**Free OH**	**3745.76**	**0–H alcohol**	**3745.76**	**O–H alcohol**
3338.78	**OH intermolecular**	**3331.07**	**O–H intermol.bonded**	**3282.84**	**C–H strech alkyne**
3288.63	**OH intermolecular**	**2924.09**	**0–H intramol.bonded**	**2852.72**	**Aldehyde doublet**
2160.27	**Azide N = N = N**	**2856.58**	**0-H intramol.bonded**	**1635.64**	**C = N imine/oxime**
2031.04	**Allene C = C = C**	**1639.49**	**C = C monosub alkene**	**1450.47**	**C-H, methyl alkane**
1633.71	**C = C monosubstituted. alkene**	**1566.2**	**C = C cyclic alkene**	**1425.4**	**O–H carboxylic**
1550.77	**C–O aliphatic ether**	**1548.84**	**N–O nitro compound**	**1396.46**	**O–H phenol**
1452.4	**C–H alkane**	**1159.22**	**Hydrate**	**1382.96**	**C–H gem dimethyl**
1396.46	**C–H aldehyde**	**1055.06**	**Primary alcohol**	**1317.38**	**O–H phenol**
1251.8	**C–N amine**			**1242.16**	**C–O aliphatic ether**
1076.28	**S = O sulfoxide**			**1055.06**	**C–O primary alcohol**

**Table 3:** Cell viability of A549 and HEK293 cells after treatment with leaf extracts and AgNPs

Sample name	Cell viability (%)	
	**A549 cell line**	**HEK293 cell line**
Control	100	100
Neem leaf	64.83	98
Madhunashini leaf	68.24	99
Moringa leaf	58.88	98
Neem AgNPs	49.64	97
Madhunashini AgNPs	63.94	96
Moringa AgNPs	50.75	97

### Scanning electronic microscopy (SEM) techniques

**Fig. 2:**
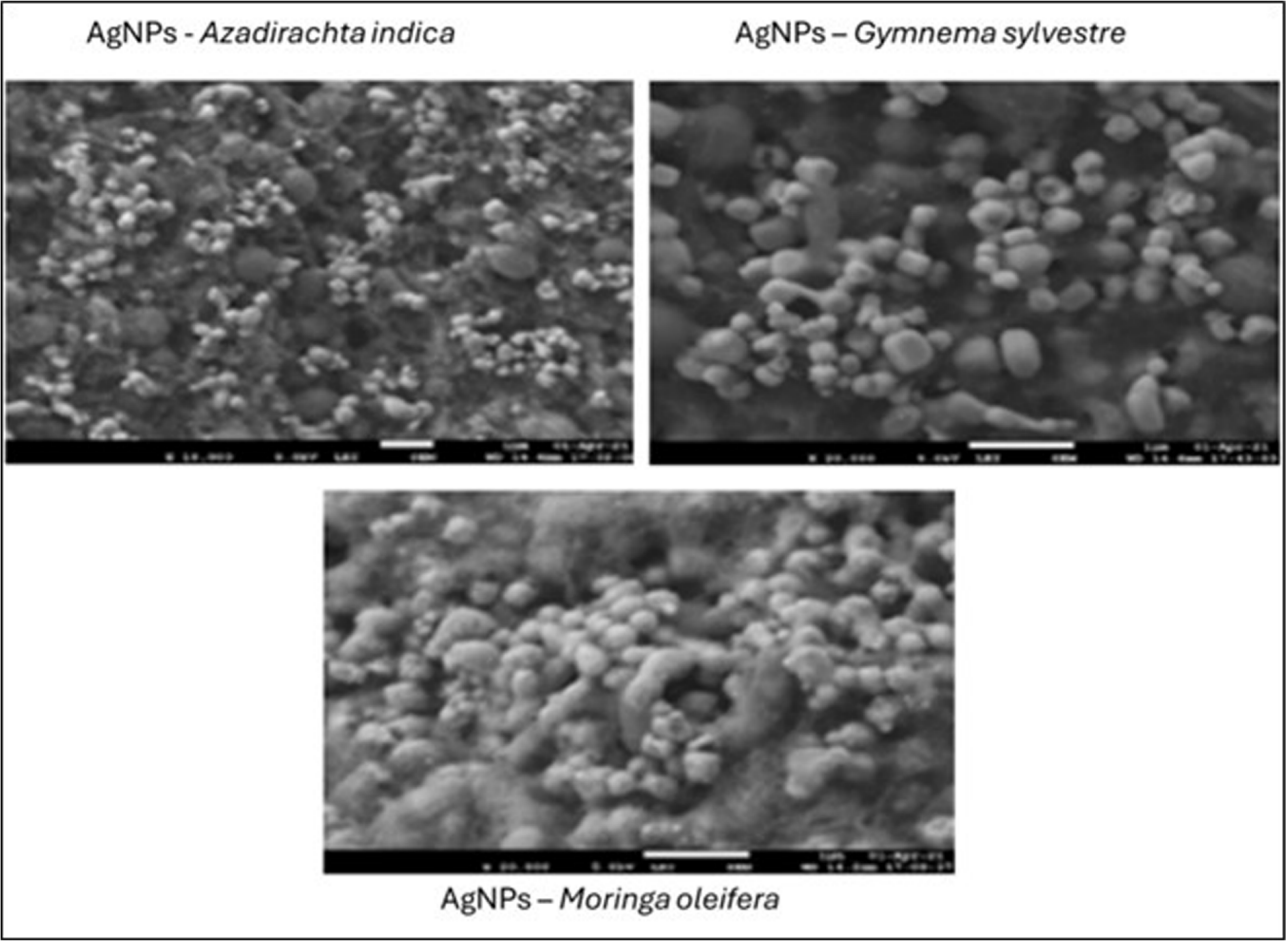
SEM images of AgNP’s of *Azadirachta indica*, *Gymnema sylvestre*, and *Moringa oleifera*

The predominant morphology of the silver nanoparticles exhibited a combination of spherical and cuboidal shapes [15, 34]. These nanoparticles underwent a bio-reduction process and were subsequently analyzed to determine their morphology, sizes, and distribution within an aqueous suspension. The preparation technique involved depositing the suspension onto a pristine glass plate and subsequently allowing complete evaporation of water, likely resulting in the deposition of a residue of nanoparticles suitable for further analysis (Fig. 2).

### X-ray diffraction studies (XRD)

**Fig. 3:**
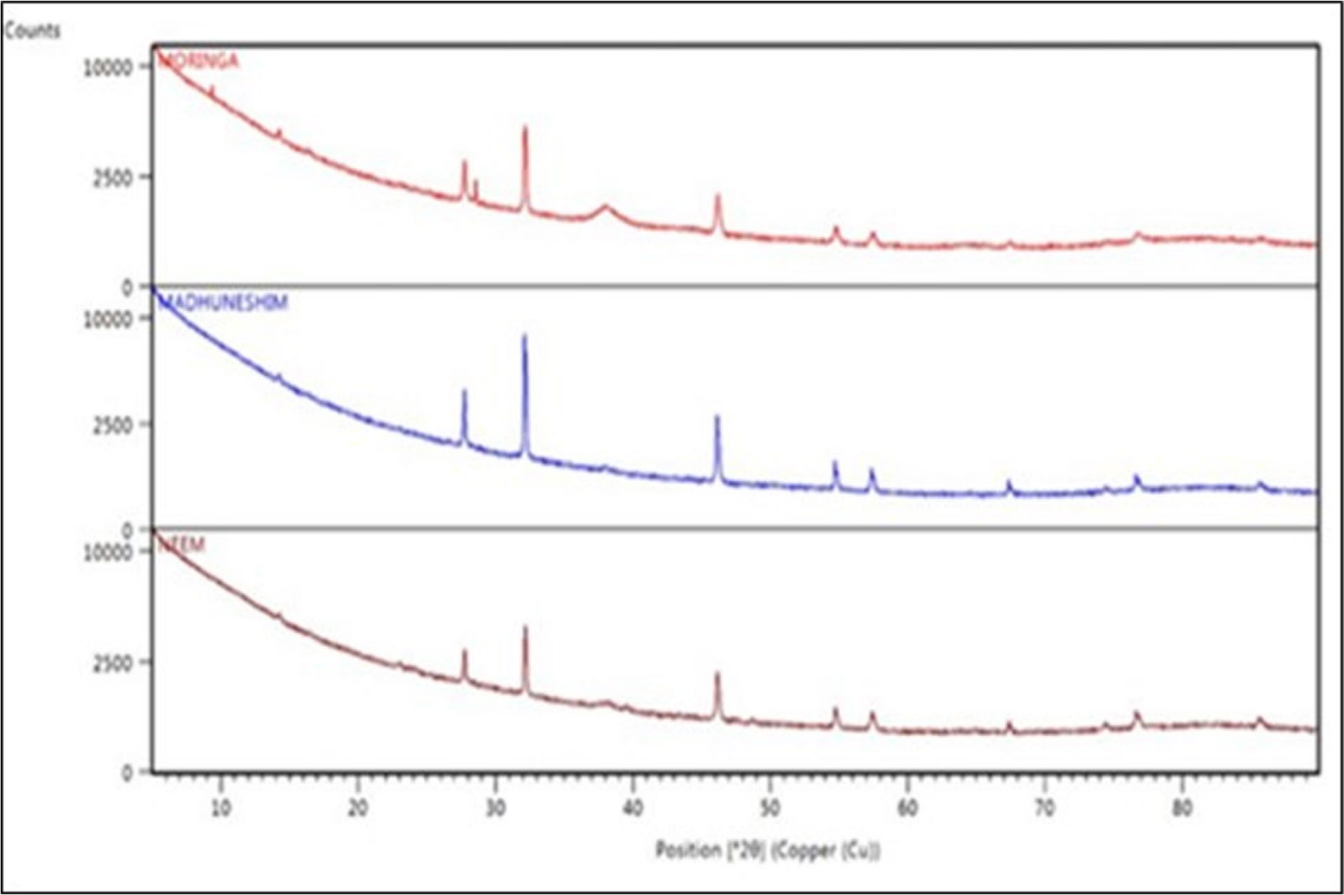
X-ray diffraction studies

XRD measurements were conducted on a thin film of silver nanoparticles. The thin film was prepared by drop-coating an aqueous solution onto a glass slide. The XRD analysis utilized a PANalytical instrument with a Cu anode [14, 15, 34]. The diffraction pattern was recorded using Cu-Kα1 radiation with a wavelength of approximately 1.54 Å, detected by a PIXcel 3D detector (Fig. 3).

The presence of sharp peaks in the XRD pattern indicates the crystalline nature of the silver nanoparticles [14, 15]. The observed peaks at specific angles correspond to the crystallographic planes of the silver nanoparticles. Here is the interpretation of the peaks identified:

- Peak at 28° corresponds to the (211) plane.
- Peak at 32° corresponds to the (113) plane.
- Peak at 38° corresponds to the (111) plane.
- Peak at 48° corresponds to the (200) plane.

These peak positions enable the identification of the crystallographic phases present in the sample. By applying Scherrer’s equation, which relates the broadening of XRD peaks to the size of the crystalline domains, the size of the nanoparticles can be calculated [15, 34]. This method provides valuable insights into the size and crystallinity of the nanoparticles in the thin film sample.

### Fourier transform infrared spectroscopy (FTIR spectroscopy)

The utilization of Fourier-transform infrared spectroscopy (FTIR) in examining synthesized silver nanoparticles using leaf extracts has proven highly beneficial [14, 15]. The dried sample was meticulously ground alongside potassium bromide (KBr) pellets and subsequently subjected to analysis using an IRAffinity-1S spectrometer (Shimadzu). Operating at a resolution of 4 cm^−1^ within the range of 4000–400 cm^−1^, the spectrometer effectively aided in identifying the biomolecules responsible for the reduction and efficient stabilization of the silver nanoparticles.

**Fig. 4:**
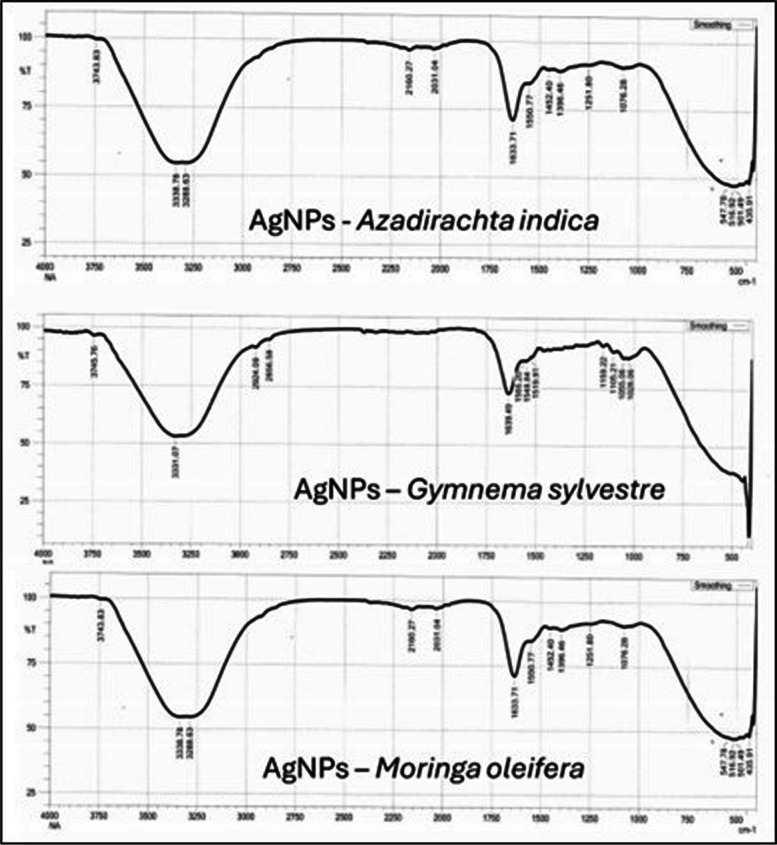
Fourier transform infrared spectroscopy analysis of AgNPs of *Azadirachta indica*, *Gymnema sylvestre*, and *Moringa oleifera*

The distinctive absorption bands observed correspond to various functional groups found in the leaf extract, such as hydroxyl (OH), carbonyl (C = O), carboxyl (COOH), and amino (NH) groups, among others [15]. The presence of these functional groups suggests the participation of specific biomolecules like phenols, flavonoids, proteins, and sugars in the reduction and stabilization of the silver nanoparticles. Through FTIR analysis, a comprehensive understanding of the chemical composition of the leaf extract and its pivotal role in the synthesis and stabilization of the silver nanoparticles was attained, thereby significantly contributing to the advancement of knowledge in these processes (Fig. 4).

### MTT or cytotoxicity assay

The MTT assay was employed to evaluate the cell viability of A549 cells treated with leaf extracts and silver nanoparticles derived from *Azadirachta indica*, *Gymnema sylvestre*, and *Moringa oleifera* at a concentration of 20 µg/ml [12, 13, 16]. This assay, commonly utilized, assesses cell viability based on the capacity of viable cells to reduce a tetrazolium dye (MTT) to formazan crystals. Following a 36-h incubation period, the percentage of cell viability was calculated for each treatment group in comparison to untreated cells (control). The findings reveal varying degrees of inhibition in the growth of A549 cells due to the treatments. The percentages of cell viability for A549 cells treated with each concentration of leaf extracts and silver nanoparticles are as follows:

- *Azadirachta indica* leaf extract and AgNPs: 64.83%, 49.64%
- *Gymnema sylvestre* leaf extract and AgNPs: 68.24%, 63.94%,
- *Moringa oleifera* leaf extract and AgNPs: 58.88%, 50.75%

**Fig. 5:**
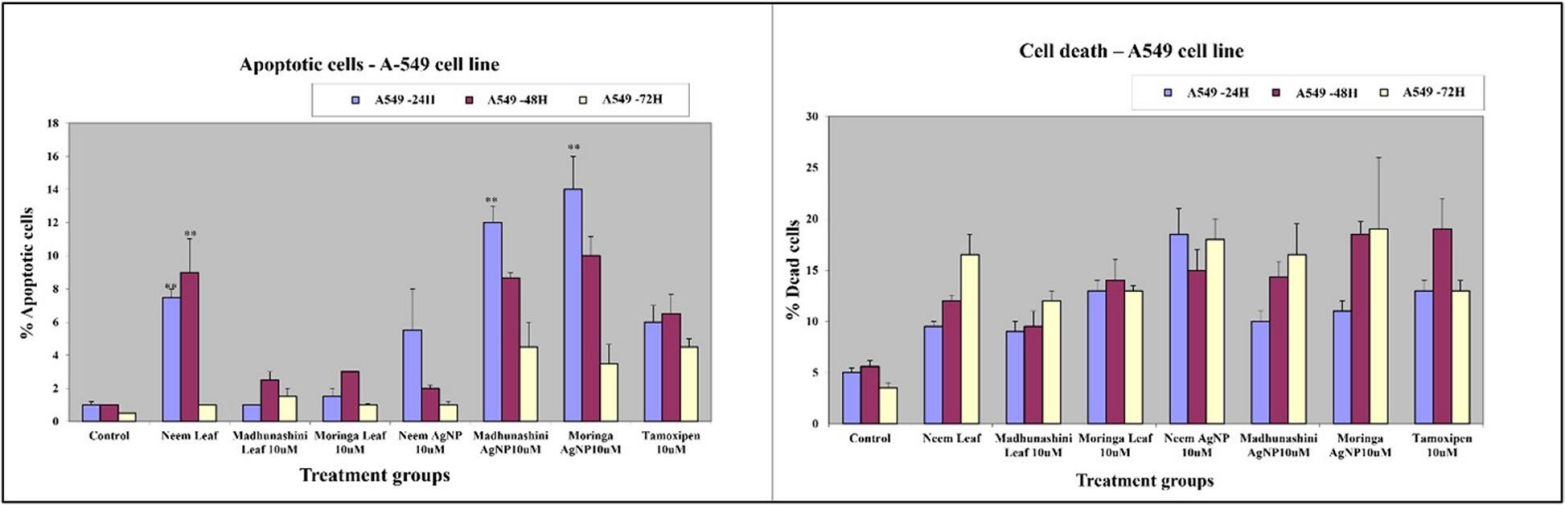
Effects of potential anti-cancer agents on cell viability and apoptosis

These outcomes imply that the treatments exert inhibitory effects on the growth of A549 cells when compared to the untreated control [12]. The disparities in cell viability among the treatments may indicate differences in the cytotoxic effects of the various leaf extracts and their silver nanoparticles on A549 cells [25]. Whereas similar treatments on HEK293 cell lines did not show any signs of cell mortality or death, indicating that these experimental treatments are cytotoxic to only cancerous cell lines [13, 25] (Fig. 5).

### Cell death and apoptosis assay

- Control Group: Represents untreated cells, serving as a baseline for comparison. The control group displays typical cellular morphology and low levels of apoptosis, as indicated by the minimal presence of Annexin V and PI-positive cells.
- Treatment Group 1 (leaf extracts): Cells treated with Leaf extracts demonstrate a significant increase in apoptotic or necrotic cells (Annexin V + /PI +) compared to the control group. This indicates that leaf extracts effectively induces apoptosis, highlighting its potential as an anti-cancer agent. Among the leaf extracts, Neem leaf extracts showed more potency (Cell death and Apoptosis), and its potency increased with exposure time.
- Treatment Group 2 (AgNPs): Similarly to the leaf extracts, Madhunashini and Moringa AgNPs showed also potency (Cell death and apoptosis) but decreased with exposure time. However, the apoptotic ability of Madhunashini and Moringa AgNPs showed also potency (cell death) with exposure time.

The figure illustrates the impact of two potential anti-cancer agents on cell viability and apoptosis. The control group shows low baseline apoptosis, while both treated groups (Leaf extracts and AgNPs) exhibit increased levels of apoptosis, as evidenced by the higher proportion of Annexin V and PI-positive cells. The data suggest that both agents can effectively induce programmed cell death in cancer cells. Notably, the distinct profiles of apoptosis in Treatment Groups 1 and 2 imply potential differences in the mechanisms of action or potency of the different agents. This observation underscores the therapeutic potential of these compounds and the need for further studies to elucidate their mechanisms and optimize their anti-cancer efficacy.

### Gene expression studies

In the analysis of gene expression in A549 cells treated with leaf extracts and silver nanoparticles, several notable observations were made regarding the GAPDH, CYCLIN-D1, and VEGF genes:

GAPDH, a housekeeping gene known for its stable expression across various tissues and cells, consistently demonstrated expression in all samples treated with leaf extracts and silver nanoparticles at 10 µg/ml, indicating that the treatment did not significantly influence GAPDH gene expression. In contrast, vascular endothelial growth factor (VEGF), which plays a pivotal role in tumor angiogenesis and signaling in tumor cells, showed variable expression at the same concentration. Notably, downregulation of VEGF was observed in samples treated with Moringa leaf extract, Madhunashini leaf extract, Neem leaf extract, and their respective silver nanoparticles, suggesting a potential inhibitory effect on VEGF expression, which could impair tumor-related functions beyond angiogenesis.

**Fig. 6:**
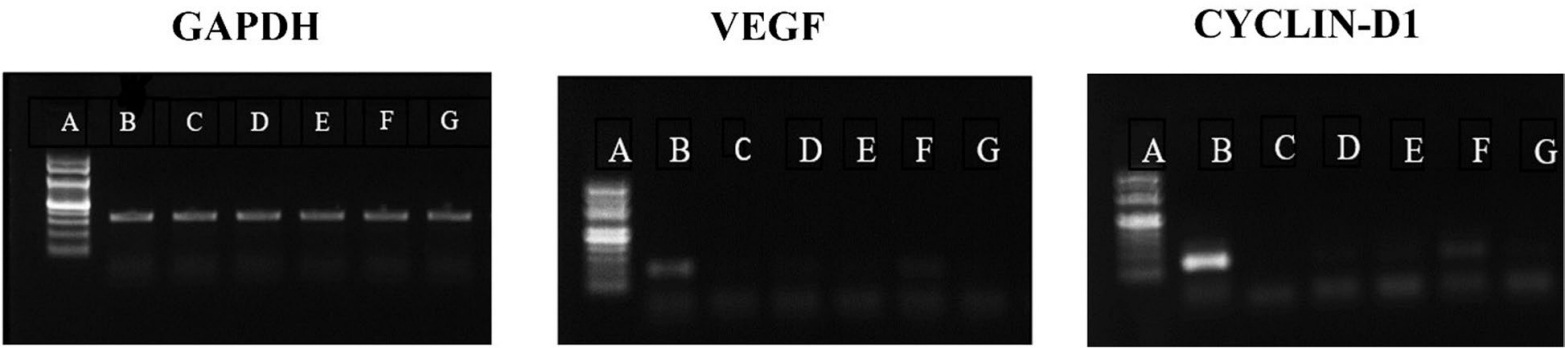
Expression studies of VEGF and CYCLIN-D1 markers in the treated samples (aqueous leaf extracts and AgNP’s). Lanes A–G: 100 bp ladder, Moringa leaf extract, Neem leaf extract, Madhunashini leaf extract, Moringa AgNP, Neem AgNP, Madhunashini AgNP

Similarly, CYCLIN-D1, a key regulator of the cell cycle and frequently overexpressed in cancer, exhibited stable expression at 10 µg/ml in most samples. However, a slight downregulation was observed in treatments with Madhunashini leaf extract, Neem leaf extract, and their corresponding silver nanoparticles, hinting at a possible inhibitory effect on CYCLIN-D1 expression. This downregulation could potentially affect cell cycle progression and reduce cancer cell proliferation. These gene expression analyses provide insights into the molecular mechanisms underlying the impact of leaf extracts and silver nanoparticles on A549 cells, particularly in terms of cell cycle regulation and tumor signaling pathways (Fig. 6).

## Discussion

The green synthesis of various metallic nanoparticles including iron, copper, gold, silver, and zinc—using medicinal plant extracts has attracted significant interest due to their diverse therapeutic potentials across a range of diseases [41–51]. For instance, iron nanoparticles synthesized from *Salvia chloroleuca* show neuroprotective effects, mitigating methadone-induced cytotoxicity and enhancing mitochondrial membrane potential in PC12 cells, suggesting potential applications in neurodegenerative disorder treatments [42]. Similarly, zinc nanoparticles derived from *Allium saralicum* exhibit strong antimicrobial, wound healing, and antioxidant properties, making them valuable for treating skin wounds and infections [43].

Silver nanoparticles (AgNPs) synthesized from *Ziziphora clinopodioides* demonstrate antidiabetic and hepatoprotective effects by significantly lowering fasting blood glucose levels and alleviating liver damage in diabetic models [44]. Gold nanoparticles synthesized from *Camellia sinensis* show promise against acute myeloid leukemia, with efficacy comparable to conventional chemotherapeutics like daunorubicin but reduced toxicity to healthy cells, positioning them as a promising alternative in chemotherapy [43–46]. Additionally, copper nanoparticles derived from *Acroptilon repens* display potent anticancer activity against lung adenocarcinoma by modulating the PI3K-Akt-mTOR signaling pathway, inducing apoptosis, and reducing cancer cell viability in a dose-dependent manner [47]. Copper nanoparticles synthesized from *Nigella sativa* seeds also demonstrate neuroprotective properties by improving cell viability, reducing inflammation, and inhibiting caspase-3 activity, thereby protecting neurons from methadone-induced damage [48]. Recent research has highlighted copper nanoparticles supported on chitosan-modified magnetic Fe₃O₄ nanoparticles as effective anticancer agents against lung cancer cell lines [49–51].

Of the various types of nanoparticles, AgNPs are particularly notable due to their small size, large surface area, and high reactivity, which are advantageous for cancer treatment. AgNPs can induce apoptosis in cancer cells by generating reactive oxygen species (ROS), damaging mitochondria, and disrupting DNA [20, 32–37]. Importantly, AgNPs demonstrate selective toxicity, targeting cancer cells more effectively than normal cells, thus minimizing side effects. They have also been shown to enhance the efficacy of conventional chemotherapy and may help overcome drug resistance in certain cancers. Additionally, AgNPs possess anti-angiogenic properties that inhibit the formation of tumor-induced blood vessels, further curtailing cancer proliferation and spread [20, 30–35]. Their ability to deliver therapeutic agents, such as gene therapies or chemotherapeutics, makes them a promising option for targeted cancer treatments [37–39].

Lung cancer remains a major global health challenge, spurring ongoing research into innovative treatments, including immunotherapy, chemotherapy, targeted therapies, and surgery [51]. The synthesis of AgNPs using extracts from *Azadirachta indica*, *Gymnema sylvestre*, and *Moringa oleifera* presents a promising approach for biomedical and pharmaceutical applications. Structural analysis through X-ray diffraction (XRD) confirms the successful production and purity of these AgNPs, supporting their therapeutic applicability [42, 43].

The green synthesis of AgNPs offers numerous advantages: it is cost-effective, as it uses plant extracts as reducing and stabilizing agents, reducing dependence on expensive reagents and energy-intensive processes. It enhances compatibility with biological systems, potentially lowering cytotoxicity and improving biocompatibility for biomedical applications, while being eco-friendly by minimizing hazardous waste and environmental impact associated with conventional chemical methods. This approach emerges as a sustainable and efficient alternative to traditional nanoparticle synthesis [40, 41].

The findings from this study underscore the potential of green-synthesized AgNPs for lung cancer treatment, specifically targeting A549 lung cancer cells. The synthesized AgNPs exhibited strong anticancer efficacy, likely due to synergistic interactions between the nanoparticles and the phytochemicals in the plant extracts, which enhance bioavailability and therapeutic impact on cancer cells. Conjugated nanoparticles demonstrated superior anticancer effects compared to aqueous leaf extracts alone, underscoring the therapeutic potential of combining phytocompounds and nanomaterials for enhanced efficacy [42–46]. Moreover, synthesizing AgNPs from plant extracts presents a sustainable route for generating nanomaterials with broad biomedical applications, addressing challenges related to cost, compatibility, and environmental impact [27–32]. The observed inhibition of A549 cancer cell viability highlights the role of secondary metabolites in these plant extracts, reinforcing the potential of green synthesis as an environmentally friendly anticancer approach [45–51].

This study further demonstrates the therapeutic potential of green-synthesized AgNPs for lung cancer treatment by leveraging phytochemicals as natural reducing and stabilizing agents. This synthesis method produces nanoparticles with optimized size, shape, and surface characteristics, enhancing cellular uptake and therapeutic efficacy [52, 53]. Surface functionalization improves AgNP biocompatibility, facilitating targeted interactions with cancer cell biomarkers while minimizing off-target effects on healthy cells [54]. Mechanistically, AgNPs promote selective cancer cell death by inducing ROS production, disrupting mitochondrial integrity, and impairing DNA structure, offering an advantage over conventional therapies [55]. When combined with existing treatments, AgNPs enhance therapeutic outcomes, particularly by overcoming drug resistance and increasing cancer cells' sensitivity to these therapies [56].

Additionally, AgNPs impact the tumor microenvironment and immune response by modulating cell signaling pathways, inhibiting angiogenesis, and enhancing immune-mediated destruction of cancer cells [57]. The bioactive compounds from medicinal plants contribute synergistic anticancer effects, potentially improving bioavailability and therapeutic impact [58]. Furthermore, AgNPs modulate gene expression, influencing non-coding RNAs and epigenetic markers that regulate cancer proliferation and apoptosis [59]. Collectively, these findings suggest that phytochemical-assisted AgNP synthesis offers a sustainable, cost-effective alternative for cancer treatment, combining plant compounds and nanoparticles to maximize efficacy while minimizing toxicity in healthy cells [60].

The increasing interest in using plant extracts as complementary and alternative therapies for lung cancer has led to investigations into the anticancer properties of plants like *Azadirachta indica* (Neem), *Gymnema sylvestre* (Madhunasini), and *Moringa oleifera*. These extracts are rich in bioactive compounds with anti-inflammatory, anticancer, and antioxidant properties. For example, *Azadirachta indica* has been studied for its role in inhibiting tumor growth and inducing cancer cell apoptosis [1]. *Gymnema sylvestre* has demonstrated the ability to induce cell cycle arrest and suppress cancer cell growth [2]. *Moringa oleifera* contains flavonoids, phenolic compounds, and other phytochemicals with established antitumor effects [3]. The findings of this study align with these observations, supporting that the use of plant-derived AgNPs and phytoextracts provides a biocompatible, sustainable approach for lung cancer treatment, advancing nanomedicine applications in oncology.

## Conclusion

The present study successfully demonstrated the eco-friendly synthesis of silver nanoparticles (AgNPs) using leaf extracts from *Azadirachta indica*, *Gymnema sylvestre*, and *Moringa oleifera*, and their significant anticancer potential against A549 lung cancer cells. The green synthesis approach not only provided an environmentally sustainable method for producing AgNPs but also leveraged the inherent bioactive compounds in these medicinal plants, which contributed to the reduction and stabilization of silver ions.

The synthesized AgNPs exhibited potent cytotoxic effects on lung cancer cells, surpassing the efficacy of the plant extracts alone. This enhanced activity can be attributed to the synergistic interactions between the nanoparticles and phytochemicals, leading to effective downregulation of key oncogenes such as VEGF and CYCLIN-D1, which are crucial for angiogenesis and cell cycle progression, respectively. Additionally, the biocompatibility of the nanoparticles was confirmed through selective cytotoxicity against cancer cells while sparing healthy cells, emphasizing their therapeutic potential.

Although this study establishes a promising framework for the development of plant-derived AgNPs as novel therapeutic agents for lung cancer treatment, further investigations are warranted. Future research should explore in-depth mechanistic studies, including epigenetic and gene expression changes induced by AgNPs, as well as preclinical trials to assess their pharmacokinetics, biodistribution, and long-term safety profiles in vivo.

In conclusion, this research paves the way for the utilization of green-synthesized AgNPs in nanomedicine, offering a cost-effective, sustainable, and potent alternative to conventional cancer therapies. By integrating the medicinal properties of *Azadirachta indica*, *Gymnema sylvestre*, and *Moringa oleifera* into nanoparticle technology, this study contributes to the evolving landscape of cancer therapeutics and holds promise for addressing the global burden of lung cancer in an eco-friendly manner.

## Future directions

Future research should focus on optimizing the synthesis of phytochemical-assisted silver nanoparticles for enhanced anticancer efficacy, investigating their mechanisms of action, validating biocompatibility in vivo, and exploring synergy with conventional therapies to address drug resistance in lung cancer treatment.

## Data Availability

No datasets were generated or analysed during the current study.
